# Mechanical power made simple: validating a simplified calculation of mechanical power in preterm lungs

**DOI:** 10.1038/s41390-024-03339-5

**Published:** 2024-06-17

**Authors:** Jack Pearson-Lemme, Ikhwan Halibullah, Tobias Becher, Hamish D. Tingay, Ellen Douglas, Monique Fatmous, Kelly R. Kenna, Prue M. Pereira-Fantini, David G. Tingay, Arun Sett

**Affiliations:** 1 Department of Paediatrics, University of Melbourne, Melbourne, VIC Australia; 2 Newborn Services, Joan Kirner Women’s and Children’s, Sunshine Hospital, Western Health, Melbourne, VIC Australia; 3 Neonatal Research, Murdoch Children’s Research Institute, Melbourne, VIC Australia; 4 Department of Anaesthesiology and Intensive Care Medicine, University Medical Centre Schleswig–Holstein, Schleswig–Holstein, Germany; 5 Department of Obstetrics, Gynaecology and Newborn Health, University of Melbourne, Melbourne, Victoria Australia

## Abstract

**Background:**

The incidence of chronic lung disease is increasing, suggesting a need to explore novel ways to understand ventilator induced lung injury (VILI) in preterm infants. Mechanical power (MP) is a unifying measure of energy transferred to the respiratory system and a proposed determinant of VILI. The gold-standard method for calculating MP (geometric method) is not feasible in the clinical setting. This has prompted the derivation of simplified equations for calculating MP.

**Objective:**

To validate the agreement between a simplified calculation of MP (MP_Simple_) and the true MP calculated using the geometric method (MP_Ref_).

**Methods:**

MP_Simple_ and MP_Ref_ was calculated in mechanically ventilated preterm lambs (*n* = 71) and the agreement between both measures was determined using intraclass correlation coefficients (ICC), linear regression, and Bland-Altman analysis.

**Results:**

A strong linear relationship (adjusted R^2^ = 0.98), and excellent agreement (ICC = 0.99, 95% CI = 0.98–0.99) between MP_Simple_ and MP_Ref_ was demonstrated. Bland-Altman analysis demonstrated a negligible positive bias (mean difference = 0.131 J/min·kg). The 95% limits of agreement were −0.06 to 0.32 J/min·kg.

**Conclusions:**

In a controlled setting, there was excellent agreement between MP_Simple_ and gold-standard calculations. MP_Simple_ should be validated and explored in preterm neonates to assess the cause-effect relationship with VILI and neonatal outcomes.

**Impact statement:**

- Mechanical power (MP) unifies the individual components of ventilator induced lung injury (VILI) and provides an estimate of total energy transferred to the respiratory system during mechanical ventilation.
- As gold-standard calculations of mechanical power at the bedside are not feasible, alternative simplified equations have been proposed.
- In this study, MP calculated using a simplified equation had excellent agreement with true MP in mechanically ventilated preterm lambs.
- These results lay foundations to explore the role of MP in neonatal VILI and determine its relationship with short and long term respiratory outcomes.

## Introduction

Worldwide, one in ten babies are born preterm.^1^ Preterm birth results in a precarious respiratory state due to lungs which are developmentally incapable of fully supporting gas exchange. Consequently, most preterm infants need artificial respiratory support, and many require mechanical ventilation via an endotracheal tube (ETT).^2^ Although often lifesaving, mechanical ventilation is a major contributor to lung injury and has long been implicated as a risk factor for the development of chronic lung disease (bronchopulmonary dysplasia, BPD). Despite advances in respiratory care, and a focus on implementing lung-protective ventilation strategies in the neonatal intensive care unit (NICU), the ongoing respiratory morbidity related to preterm birth remains high.^3^ This suggests an urgent need to better understand the impact of respiratory support strategies on lung injury after birth.

In broad terms, ventilator induced lung injury (VILI) is a result of excessive global stress and strain on the lung parenchyma.^4^ Classically, the primary contributors to VILI were proposed to be excessive pressure (barotrauma) and volume (volutrauma), and injury resulting from ventilation of collapsed alveoli (atelectotrauma).^5^ Collectively, these mechanical components are termed ergotrauma. In recent years, the concept of mechanical power (MP) has been proposed as a composite measure which unifies these individual variables and provides an overall estimate of the energy transmitted to the respiratory system over time during mechanical ventilation.

Ideally, MP is calculated using the “geometric method”. This involves mathematically integrating the pressure-volume loop measured at high resolution and multiplying the result by the respiratory rate. Although this method is the gold standard, it is not widely available clinically as the necessary calculations are not readily manageable at the bedside. Therefore, simplified equations for calculation of MP have been proposed and validated for volume-controlled ventilation.^6^ However, these equations require knowledge of the plateau pressure (P_plat_), and most preterm infants are ventilated using volume-limited, pressure-controlled ventilation (PCV) where inspiration is not held until P_plat_ is achieved. Becher et al. have proposed a simplified equation for calculation of mechanical power during pressure-controlled ventilation which does not require knowledge of P_plat_. Although this method has demonstrated good agreement with gold standard measurements in adult patients with ARDS,^7^ it has not been validated in the preterm lung.

The aim of this study is to measure the agreement between MP_Simple_ against MP_Ref_ in an animal model of the preterm lung. We hypothesise that the agreement between MP_Simple_ and true mechanical power will be sufficient so that MP_Simple_ can be used as a surrogate for calculation of mechanical power in the preterm infant population.

## Methods

### Ethics

All data utilised in this report was generated from studies in which techniques and procedures had been approved by the Animal Ethics Committee of the Murdoch Children’s Research Institute, Melbourne, Australia (A923, A940, A943, and A956) in accordance with National Health and Medical Research Council guidelines and are reported as per the ARRIVE guidelines.^8^

### Animal preparation and instrumentation

Animals were instrumented as previously described.^9–13^ 75 preterm lambs (123–129 days gestation, term gestation 140-145 days) were delivered from anaesthetised, betamethasone exposed Border-Leicester/Merino ewes via caesarean section. The fetal head was first exteriorised, the carotid vein and artery were then surgically cannulated for infusion of medications, blood sampling, and blood pressure monitoring. Following this, the lambs were intubated using a 4.0 mm cuffed ETT. The lambs were apnoeic, with spontaneous breathing suppressed from birth using ketamine and midazolam infusions.

### Physiological monitoring

Arterial blood gas analysis was performed before birth and then every 15 min throughout the experiment. At 45 or 90 min, a lethal dose of sodium pentobarbitone (100 mg/kg) was administered.^10,11,14^

### Mechanical ventilation

The lambs were ventilated using time-cycled pressure-limited (TCPL) ventilation (SLE Croydon, UK). All lambs received a fixed inspiratory time and rate with a set positive end-expiratory pressure (PEEP) and peak inspiratory pressure (PIP), without a difference in plateau pressure (P_plat_) and PIP. We intentionally selected lambs ventilated with differing TCPL settings to show that the agreement holds across different ventilator settings. Standardised approaches to ventilator settings were used based on oxygenation, carbon dioxide and the goals of each study aim, and have been published in detail before.^9,10^ The PIP and PEEP were kept constant in all groups except Group 5, and volume-targeted ventilation was used for groups 3–7. Inspiratory times were set between 0.4 and 0.5 s and an inflation pressure rise times between 0.1 and 0.4 s. Fraction of inspirated oxygen (FiO_2_), tidal volume (V_T_) and respiratory rate (RR) were titrated to target oxygen saturations (SpO_2_) of 90–95% and pH > 7.25. All lambs had spontaneous breathing suppressed from birth. The delivered ventilation measurements are shown in Table 1.

**Table 1:** Ventilation strategies.

Group	*n*	PIP (cm H_2_O)	PEEP (cm H_2_O)	T_i_ (s)	RR (bpm)	Measured V_T_ (mL/kg)	Targeted volume	Delivered Peak inflation flow (L/min)	Surfactant^a^
1	9	36.9 (3.9)	4.0 (0.0)	0.4 (0.0)	60 (0)	5.7 (2.0)	N	7.2 (1.3)	No
2	9	39.3 (1.0)	8.0 (0.0)	0.4 (0.0)	60 (0)	11.9 (2.5)	N	10.6 (1.6)	No
3	5	31.7 (1.4)	8.0 (0.0)	0.5 (0.0)	60 (0)	7.0 (0.7)	Y	4.4 (0.7)	No
4	9	31.8 (3.1)	8.0 (0.0)	0.4 (0.0)	60 (0)	6.7 (1.0)	Y	9.8 (1.1)	No
5	11	34.8 (3.3)	8.3 (0.5)	0.5 (0.0)	60 (0)	6.8 (0.9)	Y	9.8 (1.1)	No
6	14	30.7 (6.9)	8.0 (0.0)	0.5 (0.0)	48 (13)	6.2 (1.1)	Y	4.1 (0.6)	Yes
7	14	30.0 (2.9)	8.0 (0.0)	0.37 (0.03)	60 (0)	5.4 (0.9)	Y	7.7 (0.7)	Yes

All data mean (SD). Where previously published, further details of ventilation strategies can be found in listed references.

*PEEP* positive end expiratory pressure, *PIP* peak inspiratory pressure, *T*_i_ inspiratory time, *RR* respiratory rate, *V*_T_ tidal volume, *N* no, *Y* yes.

^a^200 mg/kg surfactant (Curosurf, Chiesi Pharmaceuticals, Parma, Italy) after 10 min of ventilation.

### Data acquisition

The physiological data was continuously sampled from birth at 200 Hz (Florian, Acutronic AG, Hirzel, Switzerland) to acquire the waveform data (LabChart Pro v8.1.17, AG Instruments, Sydney Australia).^9^ For each lamb, a 60 s period of waveform data at the 15 min timepoint (approximately 60 inflations per lamb) was then exported to Microsoft Excel from which MP was calculated according to both MP_Simple_ and MP_Ref_.

### Calculation of MP_Ref_ using the “geometric method”

As work is the product of force multiplied by distance, when translated to the three-dimensional motion of the lung, this equates to the total volume displaced per unit of pressure applied. Work per breath is calculated by integrating the pressure-volume loop over a breath cycle and can be converted into mechanical power by multiplication with the respiratory rate.^8^ Mathematically, the geometric method (standardised for birth weight) can be represented as:

$${{MP}}_{{Ref}}=\frac{{RR}\cdot {{\int }_{0}^{{V}_{T}}}{P}_{{aw}}{dV}}{{birth\; weight}}$$

where *V*_*T*_ is the tidal volume, *P*_*aw*_ is the airway pressure (cm H_2_O), and *V* is the volume contained in the lungs.

### Calculation of MP_Simple_

MP_Simple_ was determined using the following formula proposed by Becher et al.^10^ to calculate MP under PCV conditions.

$${{MP}}_{{Simple}}=\frac{0.098\cdot {RR}\cdot {V}_{T}\cdot (\Delta {P}_{{insp}}+{PEEP})}{{birth\; weight}}$$

where *PEEP* is the positive end-expiratory pressure (cm H_2_O), *ΔP*_*insp*_ is the change in airway pressure during inspiration (difference between peak inspiratory pressure and PEEP, cm H_2_O), *RR* is the respiratory rate, V_T_ is the tidal volume (in L) and 0.098 is the correction factor to provide results in joules/min. The simplified equation assumes a “rectangular wave” pressure waveform (a zero rise-time during inspiration), removing the requirement to calculate *T*_*slope*_.

### Statistical analysis

A minimal sample size of 58 was determined to provide a minimal acceptable reliability (intra-class correlation coefficient, ICC) of 0.8 (power = 0.8, *α* = 0.05).^12^ Data was presented as mean (standard deviation of the mean, SD) or median (interquartile range, IQR) as appropriate. The agreement of MP_Simple_ was evaluated against MP_Ref_ using an ICC with mixed effect model and linear regression analysis. Bland-Altman analysis to compare MP_Simple_ values against the MP_Ref_ values and determine bias and 95% limits of agreement (LoA).^15^ A *P* < 0.05 was considered significant. Statistical analysis was performed using R.^16^

## Results

### Demographics

Physiological data from 75 preterm apnoeic lambs from four primary study protocols (seven discrete ventilation strategies) were initially included in the study for analysis. Four lambs were excluded from the final data analysis due to insufficient breath data extraction. Clinical characteristics and blood gas analyses are detailed in Table 2. The mean (SD) MP_Ref_ measured was 1.2 (0.65) J/min·kg. MP_Ref_ ranged from 0.83 to 2.63 J/min·kg between ventilation groups (Supplementary Table 1).

**Table 2:** Animal characteristics (n = 71).

Gestational age, days	126 (1.7)	
Male, *n* (%)	40 (56%)	
Birth weight, kg	3.09 (0.46)	
Lung fluid drained, mL/kg	12.49 (6.83)	
Singletons, *n* (%)	16 (23%)	
Blood gas analysis	Fetal	15 min
pH	7.35 (0.06)	7.36 (0.11)
PaCO_2_ (mm Hg)	45.1 (6.3)	39.7 (10.3)
PaO_2_ (mm Hg)	24.8 (12.6)	40.7 (21.0)
Base excess (mmol/L)	−0.9 (3.2)	−3.0 (3.8)

All data mean (standard deviation) unless otherwise specified.

*mm Hg* millimetres of mercury, *PaCO*_*2*_ arterial carbon dioxide partial pressure, *PaO*_*2*_ arterial oxygen partial pressure.

**Fig. 1:**
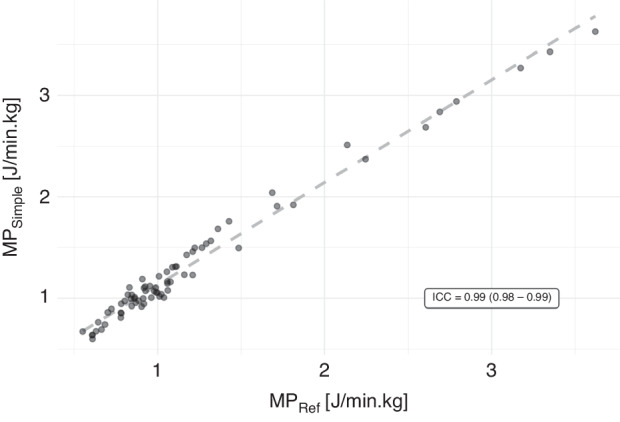
Relationship between MP values (in J/min·kg) calculated using the geometric method (MP_Ref_) and MP values calculated using the simplified MP equation (MP_Simple_). Individual dots represent individual measurements. The grey dotted line is the best fine line derived from the linear regression model. ICC intraclass correlation co-efficient, MP mechanical power.

**Fig. 2:**
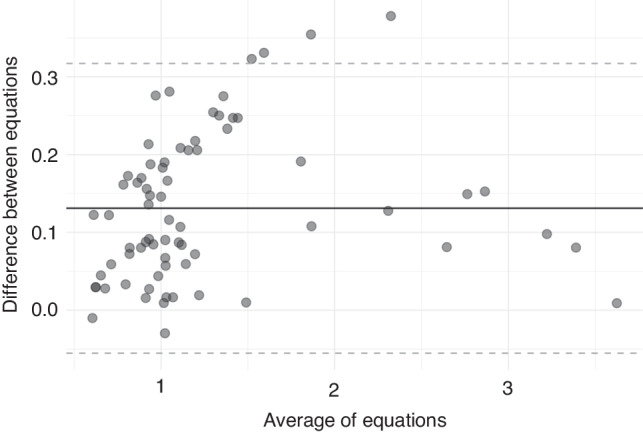
Bland-Altman plot. The average of the MP values (x-axis) is plotted against the difference between MP measurements (y-axis). The mean difference is indicated by the solid black line. Grey dashed lines represent the 95% limits of agreement. MP mechanical power.

There was excellent agreement between MP_Simple_ and MP_Ref_ (ICC = 0.99, 95% confidence interval (CI) = 0.98–0.99, Fig. 1) and the linear relationship was strong with adjusted of R^2^ = 0.98, *p* < 0.01. A Bland-Altman plot between MP_Simple_ and MP_Ref_ is presented in Fig. 2. The 95% LoAs were −0.06 and 0.32 J/min·kg. There was a mean positive bias of 0.132 J/min·kg (+11% of the average MP_Ref_). The percentage difference in positive bias varied between ventilation groups from 4 to 20%. The mean (SD) MP_Ref_, mean difference and % change, ICCs, and 95% LOAs for individual study groups are shown in Supplementary Table 1 and Supplementary Figs. 1–8.

## Discussion

Although it is understood that VILI occurs as a result of harmful mechanical states such as barotrauma, volutrauma and atelectotrauma, these components have traditionally been considered individually in the preterm lung. Such an approach is clearly flawed given the interrelationship between pressure, volume, flow, and changing disease states during mechanical ventilation. Calculation of MP as a measure of ergotrauma has the potential to unify these individual contributors of VILI. Despite being increasingly recognised in the adult critical care setting as fundamental to lung protection, there are few reports of calculation of MP in the preterm lung.^14^ Although the geometric method is the gold standard for calculating MP it has limited use in the clinical setting, as it is challenging to calculate in real time. This has prompted the derivation of simplified equations for the estimation of MP.

In this study, we analysed respiratory data from preterm lambs to assess the agreement between MP_Simple_ and MP_Ref_. We found that MP_Simple_ agrees strongly with MP_Ref_ with acceptable 95% LoAs, justifying validation studies in human infants. In addition, we utilised a formula which specifically does not require knowledge of P_plat_ and therefore is suitable for use in the neonatal population who are primarily ventilated using PCV where inspiration is not held until true P_plat_. These results provide robust evidence that a simplified calculation of MP has strong agreement with gold-standard measurements, across a variety of ventilation strategies. We have therefore concluded that MP_Simple_ is an appropriate surrogate equation for calculation of MP at the bedside.

To our knowledge, this is the first study to validate the agreement between surrogate calculations of MP and gold standard measurements in the preterm lung. Our findings are consistent with data reported from adults with ARDS.^7^ This finding is to be expected. The preterm lung shares many similar characteristics with the ARDS lung. Adult ARDS is an acute non-cardiogenic pulmonary disease that primarily occurs in patients with pneumonia, non-pulmonary sepsis, aspiration, and trauma.^13,17^ Patients with ARDS have reduced diffusing capacity and inactivated surfactant which in turn causes reduced lung compliance and refractory hypoxemia.^18^ Neonatal respiratory distress syndrome most commonly occurs due to surfactant deficiency secondary to developmental immaturity. This results in a similar pathophysiological respiratory state of low lung compliance and atelectasis. Management of both conditions is also similar, where ventilation strategies aim to reverse atelectasis using lung recruitment while avoiding excessive volume and pressure delivery. As MP has been shown to be predictive of poor outcomes in the adult ARDS population, it is plausible that calculation of MP has similar utility in preterm infants.

MP_Simple_ consistently overestimated the true MP calculated from the geometric method. This positive bias is likely due to the equation assuming a rectangular pressure waveform, ignoring the sloped inspiratory rise time over the inspiratory cycle. The positive bias has been replicated in adult and paediatric ARDS populations.^7,19^ It is possible that using low bias flow rates would lead to a greater over-estimation of MP_Simple_ due to the greater difference in total area under the curve compared to MP_Ref_. However, in our study, lambs received a bias flow that ranged between 4-11 L/min, as specified by the primary experimental protocols. Despite this range, the consistent positive bias was comparable between groups. Whether this bias is influenced by spontaneous breathing, or is clinically significant in human infants requires further study. Furthermore, the use of low bias flows as a therapeutic intervention is currently a subject of ongoing research, and not routinely used in clinical practice. Therefore, we propose that calculation of MP _Simple_ has sufficient agreement with true MP to be used in clinical trials.

There is evidence from animal and adult literature that MP may play an important role in understanding VILI and consequently patient outcomes. Cressoni et al. demonstrated in a porcine model that increases in MP were associated with increased VILI.^17^ Emerging research using MP_Simple_ has shown that higher mechanical power is associated with fewer ventilator-free days in the paediatric population with ARDS.^20^ Since MP_Simple_ has a positive bias as demonstrated in our study, the true MP likely associates even stronger with reduced mortality and morbidity. Clinically, our findings are attractive because they validate this same equation in preterm lung disease and thus facilitates the process of future research investigating the link between MP, mortality, and morbidity in preterm infants.

Mechanical power is a burgeoning area of interest and other equations for calculating MP under PCV in addition to Becher’s have been proposed. Trinkle et al. proposed an equation that additionally incorporates a prescribed rise time and flow resistance which was shown to agree even more strongly than Becher’s simplified MP equation to the reference MP in adult populations.^21^ While rise-time is not routinely manipulated in neonatal ventilation, a further study could evaluate this formula in the preterm lung against the geometric method. Furthermore, its ease-of-use should be evaluated against MP_Simple_ in the NICU clinician space.

Whilst growing in interest, the concept of MP remains novel within the preterm population.^14^ Prior to determining if MP is a significant predictor of meaningful short- and long-term outcomes, the ability to rapidly calculate MP at the bedside is required. Without scrutinising MP in interventional trials, its impact in understanding VILI in preterm infants will remain uncertain. Our findings have the potential to facilitate the incorporation of bedside calculation of MP into clinical research to better understand VILI. MP is not the only composite variable whose association with VILI is being researched. Engineering concepts such as global strain and energy load have also been shown to associate more closely with the development of VILI than individual mechanical breath parameters alone.^22^ These parameters are yet to be validated in the preterm lung but have potential in future research endeavours.

Our study has limitations. The physiological data used was obtained from apnoeic lambs which were intubated with a cuffed ETT and PEEP was limited to 4 or 8 cm H_2_O (levels consistent with current NICU practice). In clinical practice, preterm infants are usually intubated with an uncuffed ETT^23^ that results in variable degrees of cuff leak, which affects the calculation of MP. PEEP levels are similarly variable. By first calculating MP in a leak-free environment in a controlled animal model, agreement between the two MP equations could be evaluated without factoring variable cuff leak. In addition, all lambs were intubated with a size 4.0 ETT. Given that agreement between MP_Simple_ and MP_Ref_ has now been validated under controlled conditions, the next logical step will be to assess the impact of cuff leak and ETT size on the simplified MP calculation. Another important limitation of this study is the intentional suppression of spontaneous breathing. Under more clinically common circumstances, true MP will comprise of contributions from both the ventilator and changes in transpulmonary pressure and volume that occur due to spontaneous effort. This, in turn, will lead to an underestimation of MP if only ventilator data is used for calculations. Determining the agreement of MP_Simple_ and MP_Ref_ in a spontaneously breathing infant is required prior to translating these measurements into human studies, and again is the next logical step in validating MP in this population. Estimating excessive respiratory drive associated with spontaneous breathing is difficult to measure, and many of the variables used are qualitative measures or require dedicated ventilator respiratory monitoring not in routine clinical use.^24^ Indeed, patient contribution will reduce ventilator requirements, and likely lead to an underestimation in total MP calculated using only ventilator data. When applying these calculations to the spontaneously breathing infant, this limitation will require consideration.

Total MP is comprised of both the elastic and resistive work components to overcome opposing forces from the lung and chest wall.^9,25^ In our study, paired measurements were taken at a single timepoint and therefore the influence of changing lung compliance and resistance on the agreement between MP_Simple_ and MP_Ref_ could not be determined. We have previously demonstrated that surfactant administration, and therefore improving lung compliance, reduces tidal MP. The influence that therapeutic interventions and subsequent changes in lung and chest wall mechanics has on calculation of MP_Simple_ requires further study. Additionally, all lambs were mechanically ventilated, and methods to calculate MP during non-invasive ventilation require development. Finally, our study did not report the relationship between MP and VILI, although tissue was taken for analysis as part of the primary studies formulating this work. Future translational studies should consider incorporating simplified calculations of MP as an outcome measure and exploring its relationship with VILI.

## Conclusion

Simplified calculation of MP demonstrated excellent agreement with the true MP in ventilated preterm lambs. If replicated in studies in the preterm neonate, the simplified equation can easily be incorporated into NICU ventilators at the bedside for routine monitoring of MP. Further work is needed to assess whether choosing neonatal ventilation strategies that minimise MP is associated with reduced incidence of VILI and improved neonatal outcomes.

## Supplementary information

Supplementary figure table

Supplementary information

## Data Availability

Data collected during the study and the statistical analysis will be available beginning 3 months and ending 23 years after article publication to researchers who provide a methodologically sound proposal with approval by an independent review committee. Data will be available for analysis to achieve aims in the approved proposal. Proposals should be directed to arun.sett@mcri.edu.au; to gain access, data requestors will need to sign a data access or material transfer agreement approved by the Murdoch Children’s Research Institute.
