# Supplementary material for: Mechanical power made simple: validating a simplified calculation of mechanical power in preterm lungs
Source: Pediatr Res. 2024 Jun 17;97(1):178–83. doi: 10.1038/s41390-024-03339-5 (PMC11798860; doi:10.1038/s41390-024-03339-5)
Supplement: Supplementary file 1 — Supplementary figure table [file 41390_2024_3339_MOESM1_ESM.pdf]

Mechanical Power Made Simple: Validating a Simplified Calculation of Mechanical Power in Preterm Lungs: Supplementary materials

Jack Pearson-Lemme, Ikhwan Halibullah, Tobias Becher, Hamish D Tingay, Ellen Douglas, Monique Fatmous, Kelly R Kenna, Prue M Pereira-Fantini, David G Tingay, Arun Sett.

Supplementary Results

Group	Mean (SD) MP_{Ref} (J/min·kg)	Mean difference (J/min·kg)	Mean difference (% change)	ICC (95% CI)	Lower 95% LOA	Upper 95% LOA
1	0.95 (0.28)	0.06	6	0.94 (0.79-0.99)	-0.15	0.27
2	2.63 (0.72)	0.10	4	1.00 (0.98-1.00)	-0.02	0.22
3	0.98 (0.20)	0.20	20	0.96 (0.69-1.00)	0.09	0.31
4	1.02 (0.11)	0.11	11	0.96 (0.86-0.99)	-0.07	0.28
5	1.12 (0.26)	0.23	20	0.98 (0.94-1.00)	0.13	0.33
6	1.04 (0.45)	0.17	16	0.98 (0.95-0.99)	-0.01	0.36
7	0.83 (0.13)	0.07	8	0.96 (0.88-0.99)	-0.00	0.15

Supplementary Table 1: Mean (SD) MP_{Ref} per study group with corresponding, mean difference and % change, intraclass correlation coefficients (ICC), and lower and upper 95% limits of agreement for individual study groups. CI; confidence interval, LOA; limit of agreement.

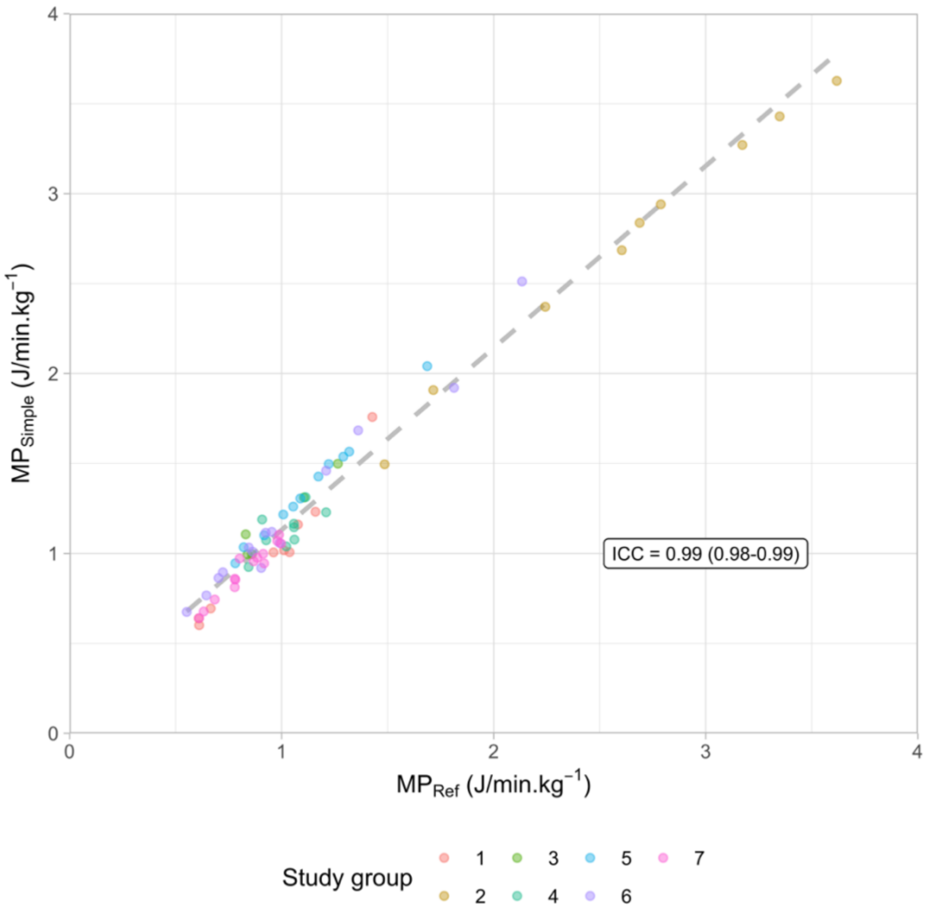

Supplementary Figure 1: Individual group relationship (individual colours) between MP values (in J/min·kg^{-1}) calculated using the geometric method (MP_{Ref}) and MP values calculated using the simplified MP equation (MP_{Simple}). Individual dots represent individual measurements. The grey dotted line is the best fine line derived from the linear regression model. ICC; intraclass correlation co-efficient.

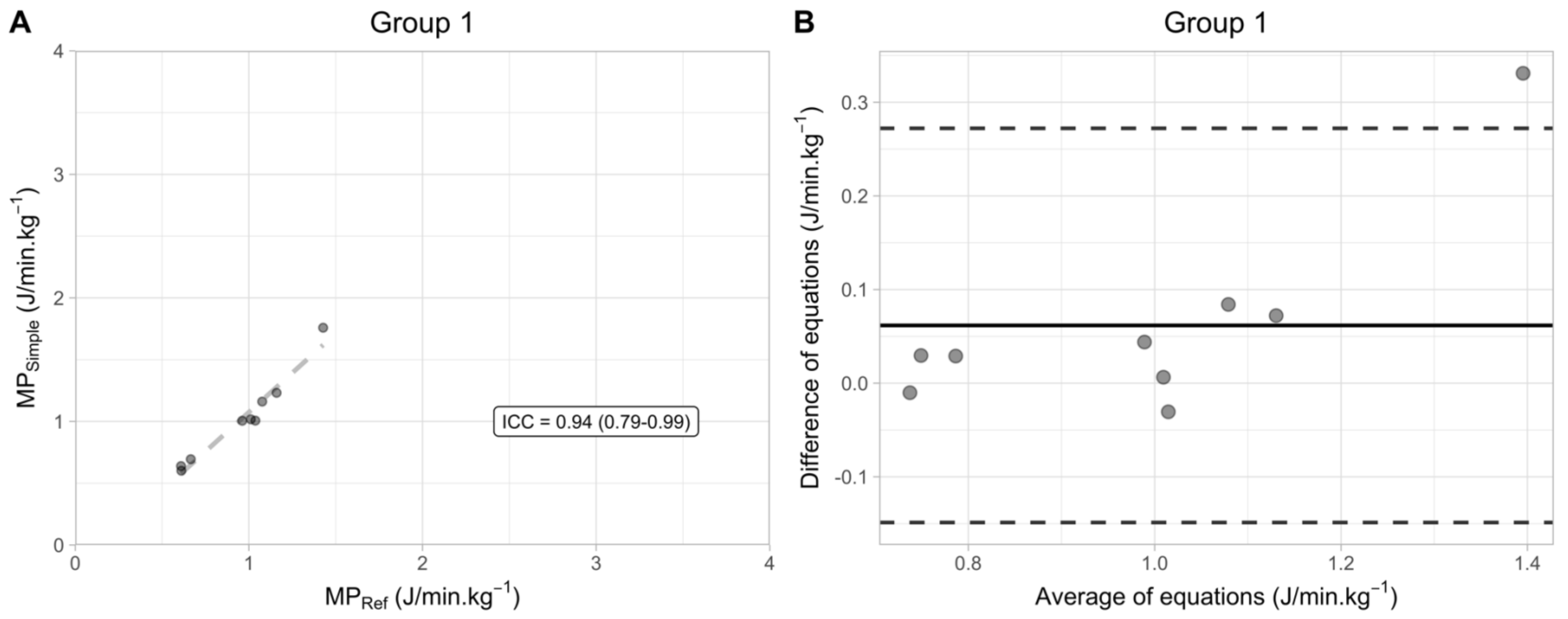

Supplementary Figure 2. (A) Scatter plot for agreement between MP_{Ref} (x-axis) and MP_{Simple} (y-axis) for Group 1. The ICC is 0.94 (0.79-0.99). **(B)** Bland-Altman plot for Group 1. The average of the MP values (x-axis) is plotted against the difference between MP measurements (y-axis). The mean difference is indicated by the solid black line (0.06 J/min.kg^{-1}). Grey dashed lines represent the 95% limits of agreement (-0.15,0.27 J/min.kg^{-1}). MP; mechanical power.

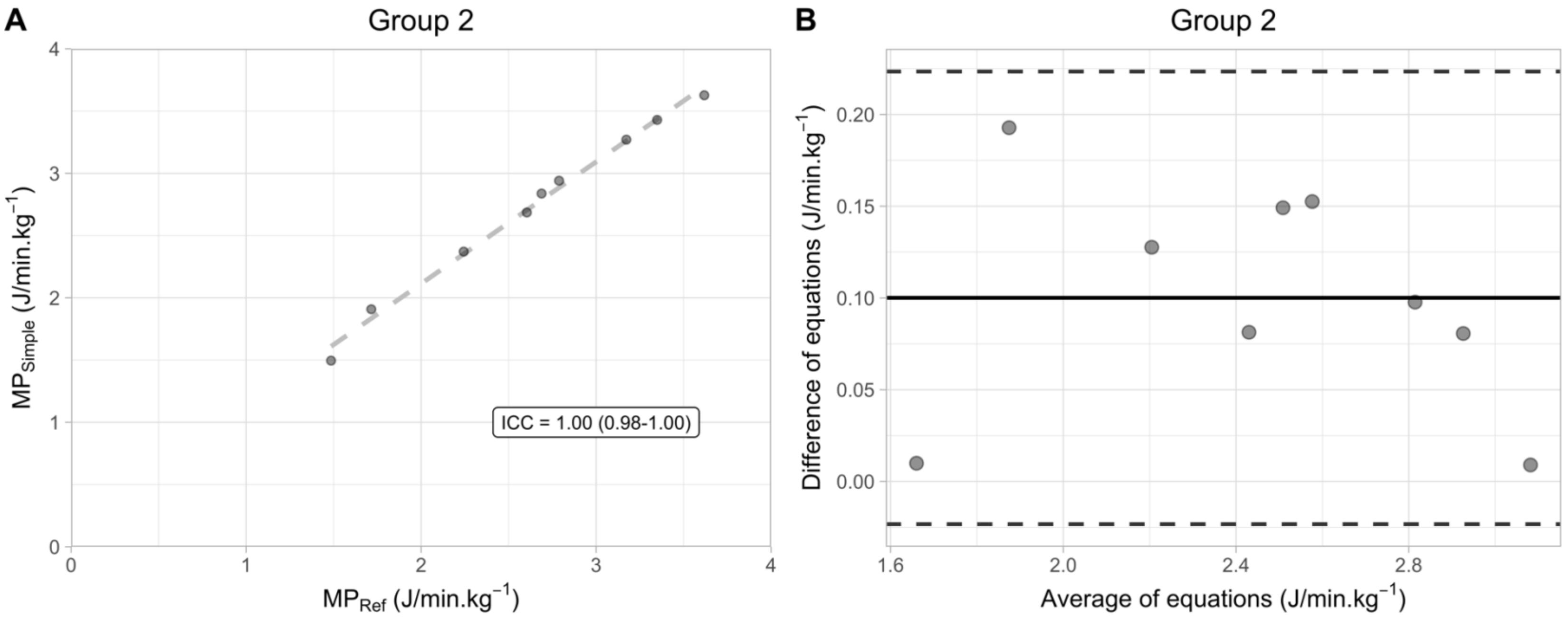

Supplementary Figure 3. (A) Scatter plot for agreement between MP_{Ref} (x-axis) and MP_{Simple} (y-axis) for Group 2. The ICC is 1.00 (0.98-1.00). **(B)** Bland-Altman plot for Group 2. The average of the MP values (x-axis) is plotted against the difference between MP measurements (y-axis). The mean difference is indicated by the solid black line (0.10 $J/min.kg^{-1}$). Grey dashed lines represent the 95% limits of agreement (-0.02,0.22 $J/min.kg^{-1}$). MP; mechanical power.

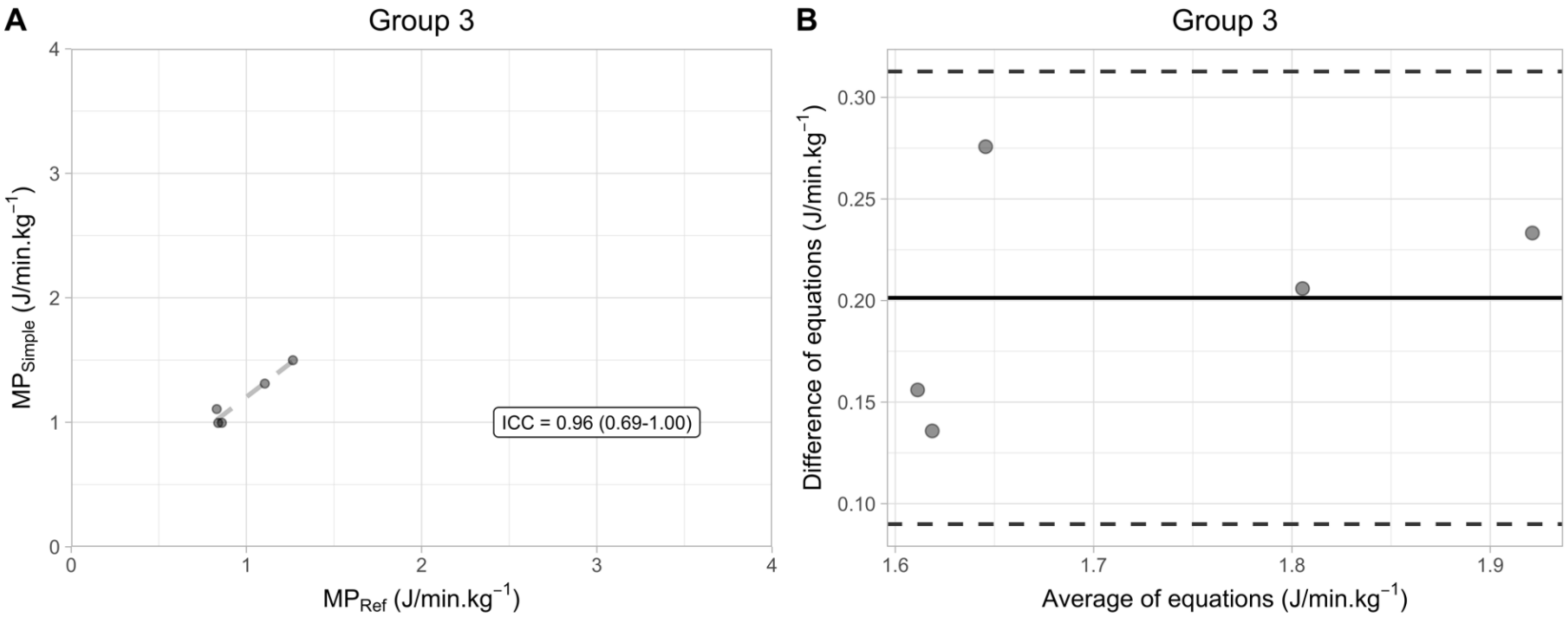

Supplementary Figure 4. (A) Scatter plot for agreement between MP_{Ref} (x-axis) and MP_{Simple} (y-axis) for Group 3. The ICC is 0.96 (0.69-1.00). **(B)** Bland-Altman plot for Group 3. The average of the MP values (x-axis) is plotted against the difference between MP measurements (y-axis). The mean difference is indicated by the solid black line (0.20 J/min.kg^{-1}). Grey dashed lines represent the 95% limits of agreement (0.09,0.31 J/min.kg^{-1}). MP; mechanical power.

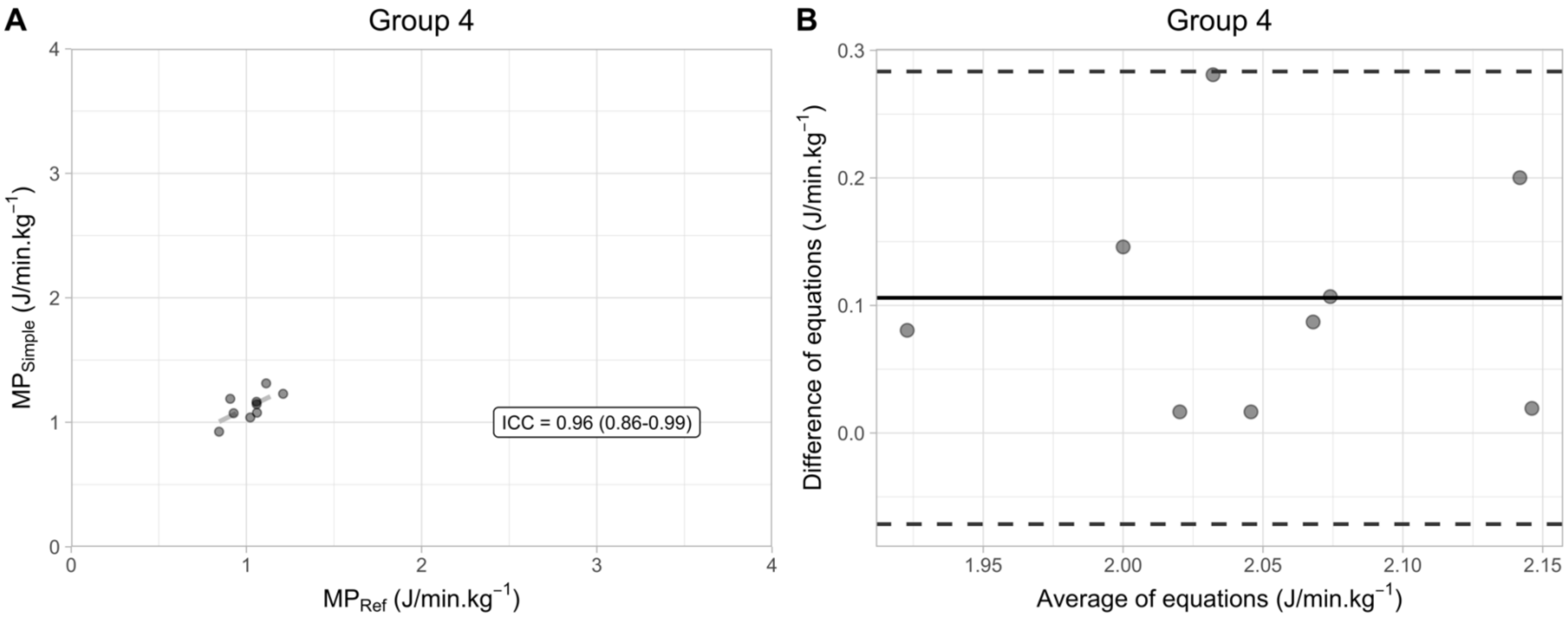

Supplementary Figure 5. (A) Scatter plot for agreement between MP_{Ref} (x-axis) and MP_{Simple} (y-axis) for Group 4. The ICC is 0.96 (0.86-0.99). **(B)** Bland-Altman plot for Group 4. The average of the MP values (x-axis) is plotted against the difference between MP measurements (y-axis). The mean difference is indicated by the solid black line (0.11 J/min.kg^{-1}). Grey dashed lines represent the 95% limits of agreement (-0.07,0.28 J/min.kg^{-1}). MP; mechanical power.

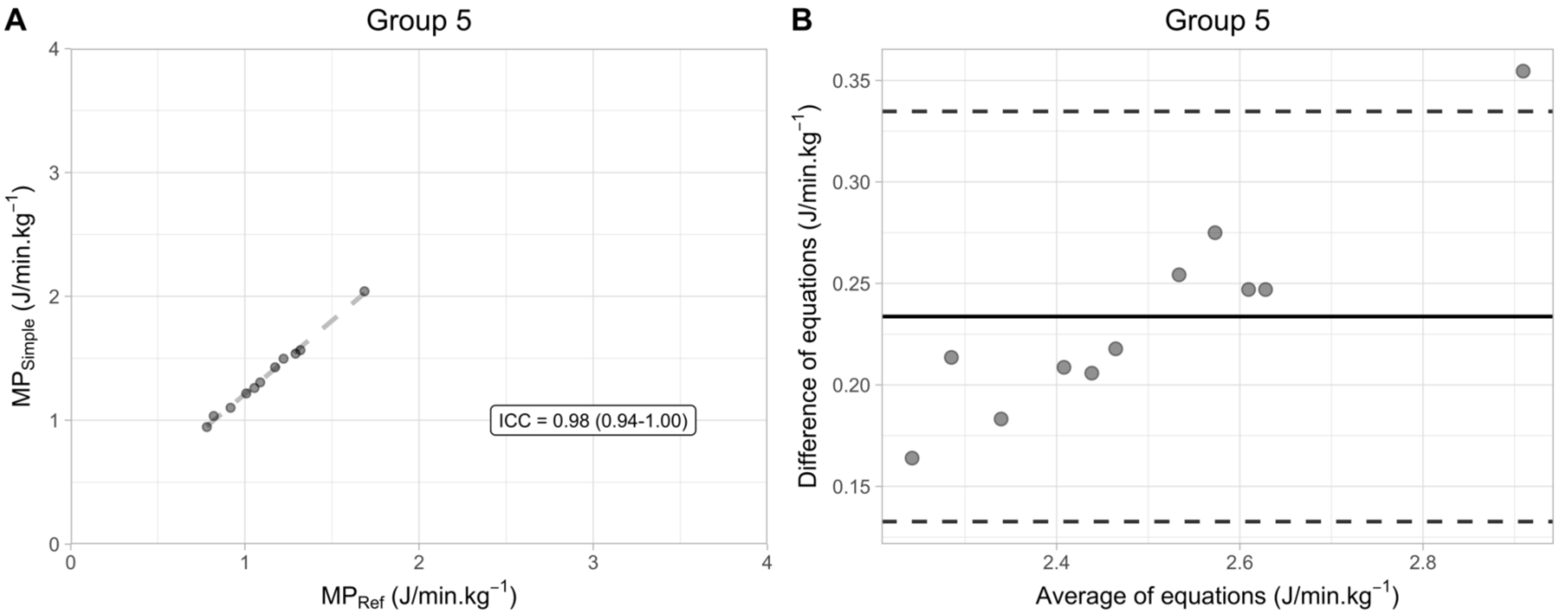

Supplementary Figure 6. (A) Scatter plot for agreement between MP_{Ref} (x-axis) and MP_{Simple} (y-axis) for Group 5. The ICC is 0.98 (0.94-1.00). **(B)** Bland-Altman plot for Group 5. The average of the MP values (x-axis) is plotted against the difference between MP measurements (y-axis). The mean difference is indicated by the solid black line (0.23 $J/min.kg^{-1}$). Grey dashed lines represent the 95% limits of agreement (0.13,0.33 $J/min.kg^{-1}$). MP; mechanical power.

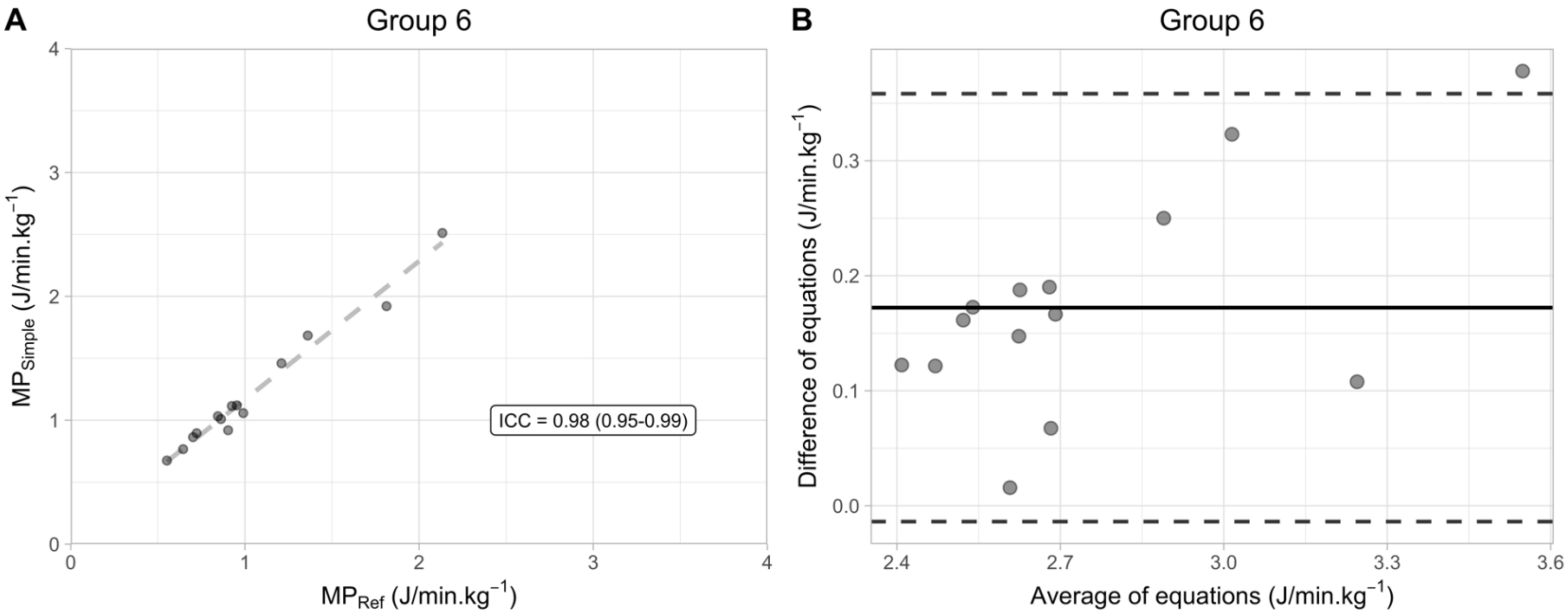

Supplementary Figure 7. (A) Scatter plot for agreement between MP_{Ref} (x-axis) and MP_{Simple} (y-axis) for Group 6. The ICC is 0.98 (0.95-0.99). **(B)** Bland-Altman plot for Group 6. The average of the MP values (x-axis) is plotted against the difference between MP measurements (y-axis). The mean difference is indicated by the solid black line (0.17 $J/min.kg^{-1}$). Grey dashed lines represent the 95% limits of agreement (-0.01,0.36 $J/min.kg^{-1}$). MP; mechanical power.

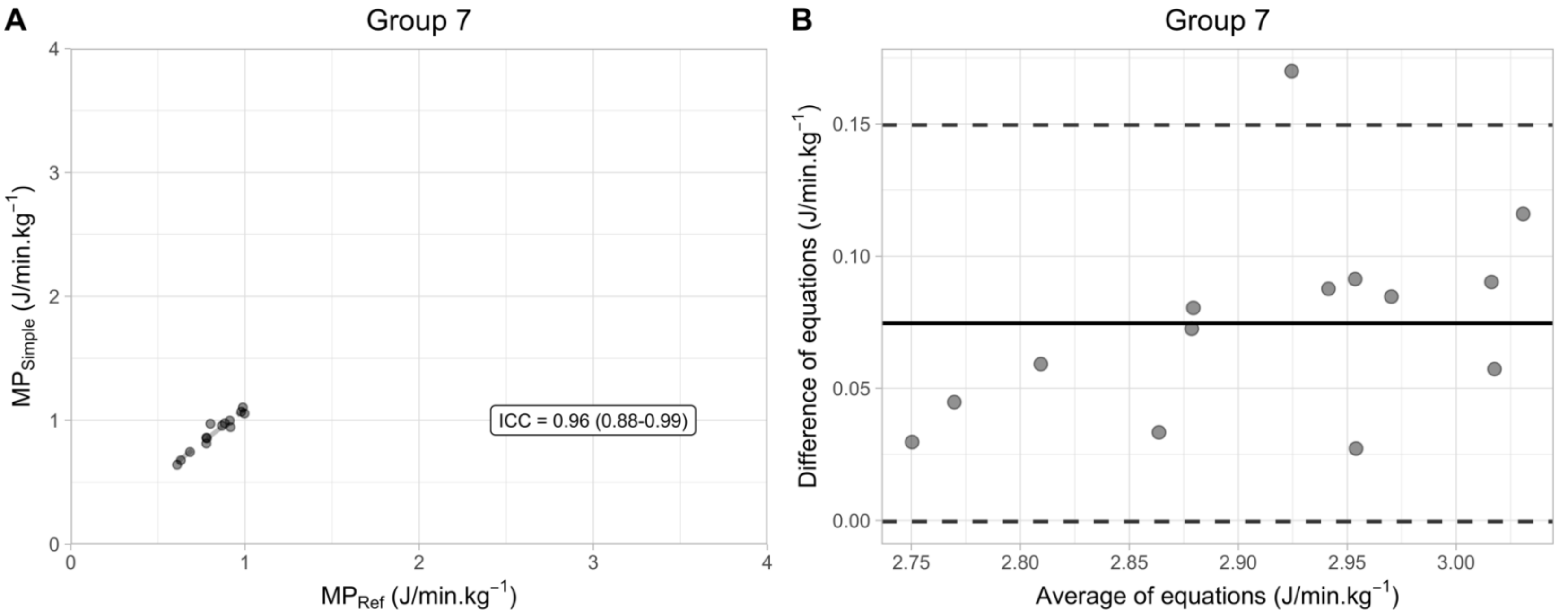

Supplementary Figure 8. (A) Scatter plot for agreement between MP_{Ref} (x-axis) and MP_{Simple} (y-axis) for Group 7. The ICC is 0.96 (0.88-0.99). **(B)** Bland-Altman plot for Group 7. The average of the MP values (x-axis) is plotted against the difference between MP measurements (y-axis). The mean difference is indicated by the solid black line (0.07 J/min.kg^{-1}). Grey dashed lines represent the 95% limits of agreement (-0.00,0.15 J/min.kg^{-1}). MP; mechanical power.
